# A familial case of VACTERL association with co-occurring sacrococcygeal teratoma: a case report

**DOI:** 10.1093/omcr/omaf046

**Published:** 2025-05-28

**Authors:** Erum Ilyas, Muhammad Affan, Razia Iftikhar, Khabab Abbasher Hussien Mohamed Ahmed

**Affiliations:** Department of Obstetrics and Gynecology, Sir Syed Medical College for Girls and Hospital, R3HH+QM9, Qayyumabad Rd, A Area Qayyumabad, Karachi, Karachi City, Sindh 75640, Pakistan; Department of Internal Medicine, Jinnah Sindh Medical University, V22W+F2H Rafiqi H Shaheed Road, Karachi, Sindh, Pakistan; Department of Obstetrics and Gynecology, Sir Syed Medical College for Girls and Hospital, R3HH+QM9, Qayyumabad Rd, A Area Qayyumabad, Karachi, Karachi City, Sindh 75640, Pakistan; Faculty of Medicine, University of Khartoum, Khartoum, Sudan 11111

**Keywords:** VACTERL association, congenital anomalies, anal atresia, tracheoesophageal fistula, sacrococcygeal teratoma

## Abstract

Background: VACTERL association is a rare disorder characterized by a non-random co-occurrence of multiple congenital anomalies. Reported incidences of VACTERL are usually sporadic.

Case Presentation: Here we present a case of familial VACTERL association in a male infant born full term at 38 weeks of gestation. The infant’s maternal aunt also had significant features of VACTERL association but died in the first year of life. Due to the lack of specialized expertise and absence of detailed anomaly scans, the congenital anomalies were not detected prenatally. The baby also had a very rare co-occurrence of sacrococcygeal teratoma.

Conclusion: Skillful personnel and equipment to detect the anomalies in-utero and the use of modern DNA sequencing techniques to detect the possible underlying genetic defects can help us better understand the pathophysiology of various congenital disorders. Early diagnosis is crucial for optimizing management strategies and counselling parents regarding potential outcomes.

## Introduction

VACTERL or VATER association is a rare, non-random co-occurrence of multiple congenital anomalies: vertebral defects, anal atresia, tracheoesophageal fistula (TEF) with esophageal atresia, and radial and renal dysplasia. The term VACTERL further includes cardiac anomalies and limb defects [1]. For the diagnosis of VACTERL association, the presence of at least three of the features is required, with TEF, limb defects, and anal atresia being more common in reported studies [2, 3]. Due to differences in diagnostic criteria and observations, the frequency has been estimated from 1 per 10 000 live births to 1 per 40 000 live births [4]. The condition itself is very rare, and its occurrence in one or more first-degree relatives is even rarer. Environmental factors including maternal diabetes, smoking, and occupational exposures, have been linked to VACTERL association [5, 6]. However, reports of a few familial incidences also hint at the possible involvement of genetic factors.

In this case report, we present a case from Pakistan of a male infant born at full term to a 27-year-old female. Multiple features of VACTERL association were observed in the infant at birth with a co-occurring sacrococcygeal teratoma. Moreover, the infant also had a family history of VACTERL association. The proband’s maternal aunt had congenital limb and heart defects and anorectal anomalies and died in the first year of life.

## Case presentation

A 27-year-old pregnant female, gravida 3 para 2, was admitted to a tertiary care hospital at 38 weeks of gestation with labor pains. The lady had been married in a non-consanguineous marriage for seven years and had no comorbidities. She had no history of tobacco, smoking, alcohol or any maternal illnesses. She was not taking any teratogenic medications. Her last two children were delivered via caesarean sections. She attended three antenatal visits at our hospital and was scheduled for a caesarean section following her first visit at 26 weeks of gestation. She already had her dating and anomaly scans done at a clinic in her locality. We did an ultrasound scan at 26 weeks of gestation and a growth scan at 33 weeks. All scans were unremarkable, with no significant findings. The pregnancy remained unremarkable throughout.

**Figure 1:**
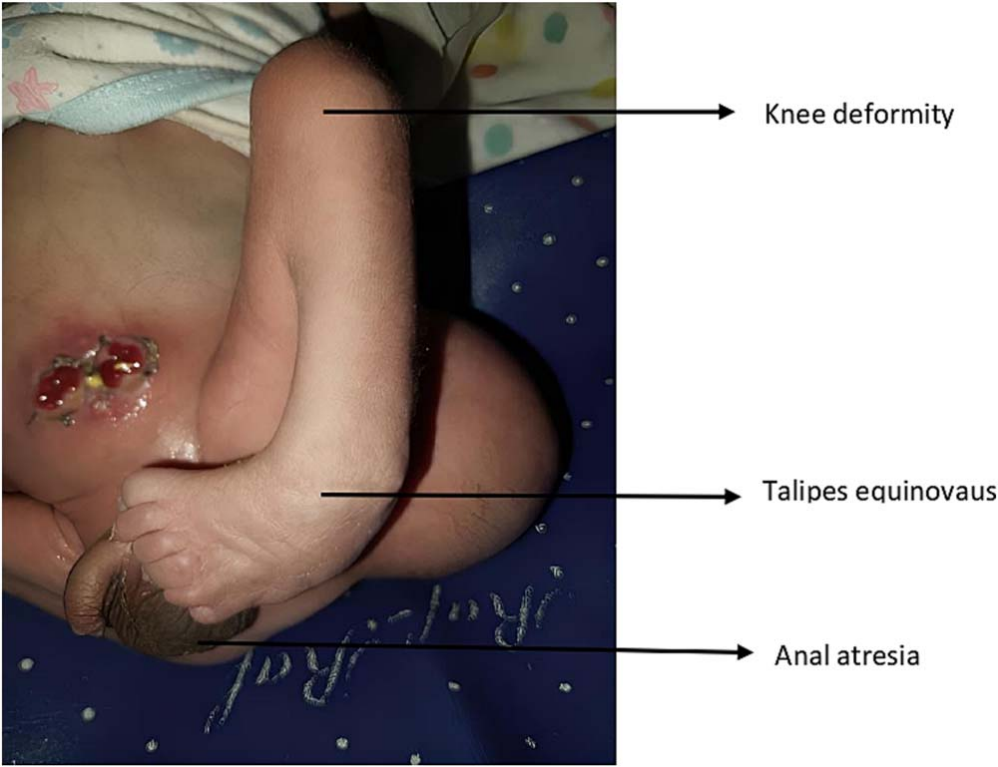
Anal atresia, deformity of left knee and talipes equinovarus.

**Figure 2:**
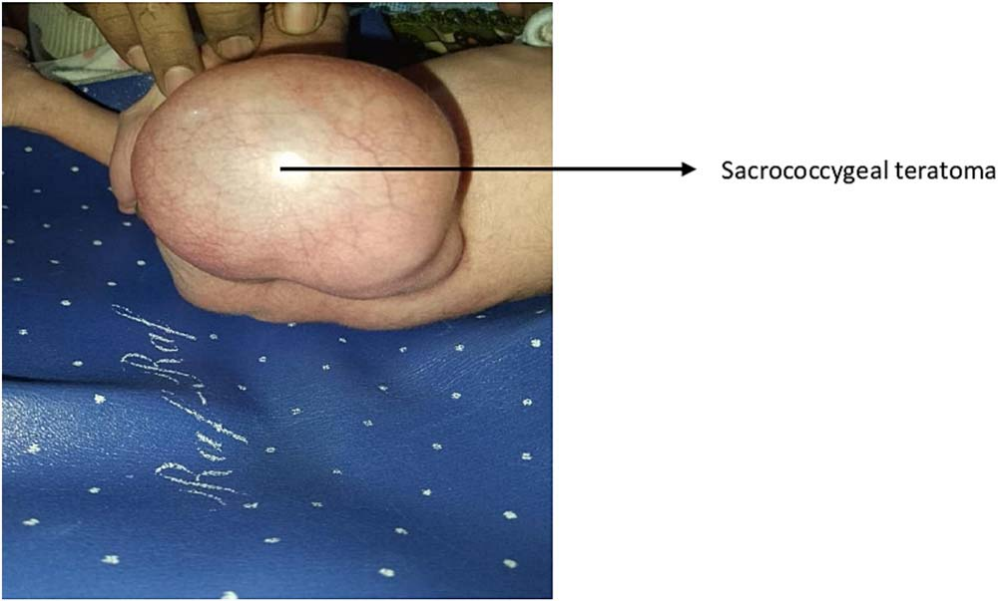
Sacrococcygeal teratoma.

The caesarean section was performed two hours after admission. An alive male baby weighing 2.7 kilograms with multiple congenital anomalies was delivered. A neonatologist diagnosed contracture deformity of the left knee, talipes equinovarus of the left foot, and imperforate anus (Figure 1: anal atresia, deformity of the left knee and talipes equinovarus). A cystic swelling on the left hip, which was provisionally diagnosed as sacrococcygeal teratoma, was also observed (Figure 2: Sacrococcygeal teratoma). The baby was referred to paediatric surgery for further management. An echocardiogram was done, which revealed a patent foramen ovale. A Kidney Ureter and Bladder (KUB) scan showed a normal right kidney; however, the left kidney could not be visualized. An MRI of the spine confirmed the presence of sacrococcygeal teratoma (Altman type 2). His first surgery was done two days after delivery, in which the sigmoid colon was brought out as a stoma. The baby was discharged after a week of surgery. The family was counselled regarding proper stoma care, hydration, diet and infection control. Additionally, the surgeons emphasized a follow-up after two weeks for further surgical plans, especially for the teratoma. The family was also counselled to return to the hospital immediately in case of an emergency and were given our ward helpline contact number. The parents were also informed that the cost of the treatment would be borne by the government at the tertiary care hospital. The parents, however, did not visit our tertiary care centre despite extensive counselling, most probably due to them belonging to a far-off rural area.

Past medical history of the patient revealed that the proband’s maternal aunt, born in the 1990s, had at least three anomalies: anal atresia, limb defects, and cardiac anomalies. The presence of these features, as per the history of the patient, could lead to the diagnosis of the VACTERL association. The female infant underwent surgery for imperforated anus but died in the first year of life due to cardiac complications.

## Discussion

VACTERL association is usually sporadic and has been proposed to be associated with a variety of environmental factors. Only a few familial cases have been reported in the past, and our case report contributes to the growing body of such cases. Solomon et al. reported an increase in the component features of VACTERL association in the first-degree relatives of patients with VACTERL than the general public using five such cases as evidence [7]. Hilger et al. in their case series presented a similar case of familial VACTERL association. In their case, they mention a female patient who displayed four characteristic features of VACTERL anomaly. Her maternal uncle also displayed four features of the anomaly [8]. Becker et al. reported two cases of a mother and her newborn son. The mother was diagnosed with a caudal duplication spectrum with associated anomalous features that significantly overlapped with the VACTERL association. The baby, on the other hand, displayed only two features of the VACTERL association [9].

VACTERL association has also been rarely reported with other congenital anomalies like sacrococcygeal teratoma. One such case was reported in our neighbouring country, India, by Gupta et al. The female infant, in that case, had tracheoesophageal fistula with esophageal atresia, anal atresia, and cardiac anomalies confirming VACTERL association. The patient also had a swelling diagnosed as Altman’s type 1 sacrococcygeal teratoma [10]. In another case report also from India, Murugasamy et.al reported a case of VACTERL association with co-existing sacrococcygeal teratoma [11]. However, we describe a unique familial occurrence of VACTERL association with a sacrococcygeal teratoma. This case emphasizes the clinical presentation of VACTERL association, other associated anomalies, and possible inheritance of gene mutations in familial cases.

Many malformations associated with VACTERL association, like renal, cardiac, and vertebral anomalies, have poor prognosis and can result in long-term complications. Therefore, VACTERL association requires extensive management of the various anomalies involved. Management usually begins with surgical treatment of life-threatening anomalies like tracheoesophageal fistula and imperforate anus. Despite advanced surgical techniques, patients face significant challenges throughout their life [4]. In addition, an associated sacrococcygeal teratoma can add to the hassle. Sacrococcygeal teratoma, although the most common congenital tumor in the newborns, is a rare disease with an incidence of 1 per 20 000–40 000 live births. Complete resection of the tumor is the standard treatment and delay in treatment can result in rupture, haemorrhage, anorectal sequelae and urological dysfunction [12]. However, associated multisystem anomalies can make it challenging to devise the best treatment plan for the patient.

VACTERL association is generally thought to be a sporadic genetic anomaly. However, reports of first-degree relatives having similar features may suggest a component of inheritance. As discussed above, some patients with VACTERL association have had first-degree relatives that had at least one feature of VACTERL association. Although all features of VACTERL might not be present, the presence of limb defects and anorectal deformities could suggest a greater likelihood of first-degree relatives of patients having VACTERL association to have one of its features than the general public. Various mutations in human genes have been found to be associated with VACTERL association, especially the renal phenotype. These include ZIC3, FGF8, FOXF, TRAP1 and LPP genes. Among these, ZIC3, present on X-chromosome, is the most frequently observed gene mutation associated with VACTERL association. Additionally, the TRAP1 gene is the first reported gene of autosomal recessive inheritance that presented with the complete clinical presentation of VACTERL anomalies [13].

Genetic testing, including Next Generation Sequencing (NGS), can be used to detect genetic variants or DNA mutations. These techniques can sequence large DNA and RNA sequences in a relatively short period. Genetic testing such as NGS can aid in the early detection of fetal anomalies and guide decisions regarding pregnancy management. Due to a lack of resources and personnel, we could not perform such tests, which can be reported as a major limitation of our study [14]. Congenital anomalies can be detected very early in pregnancy through modern techniques, as demonstrated in previous cases reported in the literature [15]. Realistic Vue imaging and Crystal Vue imaging are three-dimensional approaches that have shown remarkable potential to view the embryo in utero with great detail and depth. Fetal development, chromosomal abnormalities, and even the behaviour of the baby can be well-assessed through these advanced techniques [16, 17]. However, these techniques are currently not available at large in our country, and radiology centres that have these facilities are very expensive.

Moreover, our patient was lost to follow-up which is also a major limitation of our study. As mentioned earlier, the patient’s family belonged to a rural area far away from our tertiary care centre. Lack of follow-up limits our understanding of the prognosis of the disease and the surgical interventions done to improve the quality of life of the patient. The rural areas in our country do not have the facilities to tackle with such complex cases. The patient’s family was although counselled for further surgical interventions to improve the survival and quality of the patient, however they did not return to our hospital. The surgical treatment for the sacrococcygeal teratoma was yet to be done and the lack of follow-up may have put the patient in life-threatening danger. Improved healthcare infrastructure in rural areas is essential to facilitate timely diagnosis and management of complex congenital conditions.

Nevertheless, ultrasound scans, including anomaly scans, can also detect such an extensive set of multisystem anomalies. Our patient had been visiting a local healthcare center and visited our tertiary care center only late in her pregnancy. The rural areas of our country do not have experienced personnel and equipment that can offer good patient care. The sonologist at the regional center was not able to detect anomalies on the anomaly scan. This may or may not be due to a lack of skills or sound equipment. Moreover, the patient belonging to a rural and relatively lower socio-economical and educational background may have played a role in fewer visits to the healthcare centers and not opting for better antenatal care. Early detection of multiple anomalies can help the obstetrician to offer termination of pregnancy in the early stages. A detailed anatomical survey should be performed by an expert sonologist at 22 weeks of pregnancy. The couple should be counseled regarding the prognosis and life expectancy of the baby, depending on the severity of the condition. We also could not perform a long-term follow-up of the patient due to the family belonging to a rural town away from our tertiary care center. Further workup could not be done, and this also adds to the limitations of our study.

Management of such complex cases with a constellation of anomalies requires a multidisciplinary approach including early prenatal screening and thorough counselling. Moreover, managing familial cases can be challenging with various uncertainties. Genetic counselling can play a major role in managing such cases, especially in developing countries where not many people are well-educated and aware of the implications of supervised pregnancies, regular antenatal screening and genetic testing. Moreover, consanguineous marriages should be avoided in families where inheritance may be suspected and this further requires extensive counselling of these families. Good antenatal scans can reveal the anomalies at an early stage that can further enable the physicians to counsel the parents regarding the prognosis of the disease. Genetic testing can reveal the possible genetic mutations that may be inherited in families and can result in familial cases of such wide spectrum of anomalies.

## Conclusion

VACTERL association is a multisystem anomaly that most often occurs sporadically without a family history of the association or its component features. However, a few familial cases have also been reported that support the involvement of genetic factors. The prognosis of the VACTERL association depends on the severity of the condition, and the treatment strategies impose a considerable healthcare burden. Pre-natal diagnosis of anomalies is essential for better counseling and decision-making. The need for modern techniques and skilled personnel is significant for the proper diagnosis of these conditions. Raising public awareness about the importance of specialized prenatal care and early diagnostic evaluations at reputable healthcare centers is essential for reducing morbidity and mortality.

## Data statement and availability

All the relevant study data is available from the corresponding author upon reasonable request.
